# The Great Northern Central's Pay Wards

**Published:** 1894-12-15

**Authors:** 


### THE GREAT NORTHERN CENTRAL'S PAY WARDS.

Dr. Frank Godfrey presided over an adjourned meeting of medical practitioners resident in the North London district on Thursday week for the further consideration of the question of the pay wards which have recently been opened at the Great Northern Central Hospital. The local medical men, as is now well-known, object strongly to the pay wards as an unethical form of medical competition in which they think they are likely to come off second best. The northern practitioners recognise that they have entered upon a contest which may extend over a much wider than its present area. They see, by anticipation, pay wards, officered by honorary medical staffs, and withdrawing patients from family practice, looming large wherever a general or special hospital rears its head. They think they are fighting the battle, not of the family doctors of North London only, but of general practitioners everywhere. As one of them boldly expressed it in the simile of a famous protestant reformer of the Stuart period, they believe they are "lighting a candle of medical defence and progress which will not be easily put out."

To do the doctors justice, they approached the subject, not merely without passion, but with the dignity of cultured men. At a former meeting indignation had prevailed over cool wisdom, and a resolution had met with many ardent supporters whose purport was the stern "boycotting" of every member of the Great Northern Central's medical and surgical staff. At the meeting on Thursday soberer counsels prevailed. There were still a good many boycotters present, and one gentleman defended the principle in general, and medical boycotting in particular, in a decidedly humorous speech. He was, he said, amazed that anybody, but more especially any medical man, should avow an objection to a legitimate "boycott." Boycotting was, he affirmed, one of the oldest, one of the commonest, and one of the most honoured instruments known to the medical profession for the suppression of unethical practises. Did a man act unprofessionally? Then let him be boycotted! That was the written or unwritten rule of unvarying medical procedure; and whether the man were President of the College of Physicians or the proprietor of a shilling dispensary the rule would be rigidly applied in practice until he repented and mended his ways. The medical staff of the Great Northern Central Hospital had, he declared, entered upon a course of conduct which was not only ethically indefensible, but which was opposed to the best interests of the profession of medicine generally. That being so, he affirmed that to subject them to the action of a general "boycott" was to do to them exactly what orthodox medicine had done to unorthodox from the very beginning of medical time. The speech was well received, and tended much to the maintenance of an easy and philosophic temper throughout the discussion.

But notwithstanding the presence of a sterner element, the majority of the meeting were for mild but resolute counsels. They were determined to put a stop to the treatment of paying patients by honorary physicians and surgeons if that might be possible; but if not, then they would resolve upon some method of procedure which should bring a stern retribution upon those honorary physicians and surgeons of the Great Northern Central Hospital who, as they declared, were betraying the family practitioners into the hands of the enemy. There was always this resort, one gentleman declared, that general practitioners could call in one another if recognised consultants failed to honour the obligation to "do as they would be done by." The staff of the Great Northern Central Hospital, he said, consisted almost exclusively of young men who had still their spurs to win, and for his part he preferred as a consultant an experienced general practitioner to an inexperienced member of the staff of any hospital, Great Northern Central or otherwise. This sentiment was received with marked approbation by practically the whole of the meeting.

A resolution, which was proposed by Dr. Boulger and seconded by Dr. Beeton, was duly supported by several speakers, and met with general acceptance. The resolution affirmed that, whilst the meeting regretted the rupture of amicable relations with the staff of the Great Northern Central Hospital, it declared the conduct of the staff in its relation to the pay wards of the hospital to be ethically indefensible and opposed to the best interests of the profession of medicine. There was no desire, the mover and seconder insisted, to quarrel either with the Great Northern Central Hospital or its professional staff; but there was the sternest possible determination to resist the growing encroachments of all hospitals upon the domain of private and family practice. This determination was manifestly very strong among the younger men. The older men were milder. But the young men, some of whom spoke with marked effectiveness, were resolved, as they said, not to be ground beneath the heel of unfair and unethical medical competition any longer. They were willing to give the medical staff of the Great Northern Central Hospital time to reconsider their position; but if reconsideration did not bring about amendment, the staff must prepare itself for unpleasant consequences.

After several speeches of this kind the Chairman said he should be glad if Dr. G. W. Potter, who was present, and who knew a good deal about the whole question, would address the meeting. Dr. Potter stated that a reference to the earlier proceedings of the body which had initiated a movement for a new hospital in North London, and of which he had been the honorary secretary, would reveal the fact that the pay wards which had been originally proposed in connection with the new hospital had been designed, not for the practice of the medical staff of the hospital but solely for the convenience of the general practitioners of the neighbourhood, and for the advantage of such of their paying patients as might be living in lodgings, and consequently unable to command good nursing and attendance when they were seriously ill. Fortunately, he said, a circular bearing date 1883 had been preserved by the Chairman of the House Committee (Mr. Frazer Black, J.P.), and that circular proved in the most convincing manner that the proposed pay wards had been intended for no other purpose but the treatment of paying patients in the pay wards by their own medical attendants. Dr. Henty, who had also been a member of the original committee, said he could confirm every statement which Dr. Potter had made. Dr. Stevens, of Newington Green, and Dr. Leonard also stood up in the meeting and stated that they, too, had been members of the original committee, and could bear testimony to the exact and literal correctness of Dr. Potter's statements. The meeting was very enthusiastic and very resolute, though good tempered from commencement to close. There is no doubt that the practitioners of North London are thoroughly roused and unanimous, and are determined to fight the question out to the end. Outside sympathy was very abundant, and expressed itself in letters from Mr. Nelson Hardy and other well-known reformers of what they called "hospital encroachments and abuses."

